# The burden and predisposing factors of non-communicable diseases in Mashhad University of Medical Sciences personnel: a prospective 15-year organizational cohort study protocol and baseline assessment

**DOI:** 10.1186/s12889-020-09704-3

**Published:** 2020-11-02

**Authors:** Fariba Tohidinezhad, Ali Khorsand, Seyed Rasoul Zakavi, Reza Rezvani, Siamak Zarei-Ghanavati, Majid Abrishami, Ali Moradi, Mahmoud Tavakoli, Donya Farrokh, Masoud Pezeshki Rad, Bita Abbasi, Mitra Ahadi, Lahya Afshari Saleh, Mohammad Tayebi, Mahnaz Amini, Hossein Poustchi, Ameen Abu-Hanna, Saeid Eslami

**Affiliations:** 1 Department of Medical Informatics, Faculty of Medicine, Mashhad University of Medical Sciences, Mashhad, Iran; 2 Department of Complementary and Chinese Medicine, School of Persian and Complementary Medicine, Mashhad University of Medical Sciences, Mashhad, Iran; 3 Nuclear Medicine Research Center, Mashhad University of Medical Sciences, Mashhad, Iran; 4 Department of Nutrition, Faculty of Medicine, Mashhad University of Medical Sciences, Mashhad, Iran; 5 Eye Research Center, Mashhad University of Medical Sciences, Mashhad, Iran; 6 Department of Orthopedic Surgery, Faculty of Medicine, Mashhad University of Medical Sciences, Mashhad, Iran; 7 Department of Urology, Faculty of Medicine, Mashhad University of Medical Sciences, Mashhad, Iran; 8 Department of Radiology, Faculty of Medicine, Mashhad University of Medical Sciences, Mashhad, Iran; 9 Gastroenterology and Hepatology Research Center, Mashhad University of Medical Sciences, Mashhad, Iran; 10 Department of Occupational Medicine, Faculty of Medicine, Mashhad University of Medical Sciences, Mashhad, Iran; 11 Atherosclerosis Prevention Research Center, Mashhad University of Medical Sciences, Mashhad, Iran; 12 Lung and Tuberculosis Research Centre, Mashhad University of Medical Sciences, Mashhad, Iran; 13 Liver and Pancreatobiliary Diseases Research Center, Digestive Disease Research Institute, Tehran University of Medical Sciences, Tehran, Iran; 14 Department of Medical Informatics, Amsterdam UMC-Location AMC, University of Amsterdam, Amsterdam, The Netherlands; 15 Pharmaceutical Research Center, Mashhad University of Medical Sciences, Mashhad, Iran

**Keywords:** Non-communicable diseases, Outcome assessment, Etiology, Cohort studies, Developing countries

## Abstract

**Background:**

The rising burden of premature mortality for Non-Communicable Diseases (NCDs) in developing countries necessitates the institutionalization of a comprehensive surveillance framework to track trends and provide evidence to design, implement, and evaluate preventive strategies. This study aims to conduct an organization-based prospective cohort study on the NCDs and NCD-related secondary outcomes in adult personnel of the Mashhad University of Medical Sciences (MUMS) as main target population.

**Methods:**

This study was designed to recruit 12,000 adults aged between 30 and 70 years for 15 years. Baseline assessment includes a wide range of established NCD risk factors obtaining by face-to-face interview or examination. The questionnaires consist of demographic and socioeconomic characteristics, lifestyle pattern, fuel consumption and pesticide exposures, occupational history and hazards, personal and familial medical history, medication profile, oral hygiene, reproduction history, dietary intake, and psychological conditions. Examinations include body size and composition test, abdominopelvic and thyroid ultrasonography, orthopedic evaluation, pulse wave velocity test, electrocardiography, blood pressure measurement, smell-taste evaluation, spirometry, mammography, and preferred tea temperature assessment. Routine biochemical, cell count, and fecal occult blood tests are also performed, and the biological samples (i.e., blood, urine, hair, and nail) are stored in preserving temperature. Annual telephone interviews and repeated examinations at 5-year intervals are planned to update information on health status and its determinants.

**Results:**

A total of 5287 individuals (mean age of 43.9 ± 7.6 and 45.9% male) were included in the study thus far. About 18.5% were nurses and midwives and 44.2% had at least bachelor’s degree. Fatty liver (15.4%), thyroid disorders (11.2%), hypertension (8.8%), and diabetes (4.9%) were the most prevalent NCDs. A large proportion of the population had some degree of anxiety (64.2%). Low physical activity (13 ± 22.4 min per day), high calorie intake (3079 ± 1252), and poor pulse-wave velocity (7.2 ± 1.6 m/s) highlight the need for strategies to improve lifestyle behaviors.

**Conclusion:**

The PERSIAN Organizational Cohort study in Mashhad University of Medical Sciences is the first organizational cohort study in a metropolitan city of Iran aiming to provide a large data repository on the prevalence and risk factors of the NCDs in a developing country for future national and international research cooperation.

## Background

Non-communicable diseases (NCDs) are chronic conditions emerging as a result of a combination of genetic, physiological, environmental, and behavioral risk factors [1]. The NCDs are rarely completely curable characterized by long latency period and debilitating manifestations. In recent decades, the NCDs have been the leading cause of death worldwide, accounting for 70% (40 million in 2016) of all deaths annually [2]. A considerable proportion (37.5%) is attributable to the adult population aging between 30 and 70, which is known as premature death [2]. It is critical to note that over 80% of premature deaths occur in developing countries [2]. Furthermore, the rising global burden of NCDs has been estimated to be over $7 trillion without taking action until 2030 [2]. This will negatively affect the primary healthcare coverage in most of the middle- and low-income countries [3].

In Iran, the four main types of NCDs account for 64.5% of deaths which is higher than the global average of 57.7% (cardiovascular disorders 41.9% vs. 32.3%, cancer 14.9% vs. 16.3%, diabetes 3.9% vs. 2.6%, and respiratory disorders 3.8% vs. 6.5%) [4]. These NCDs have led to 7 million years that are lived with disability (YLDs) [4], an outcome which is considered as catastrophic health expenditure due to both productivity reduction and diversion of healthcare resources from productive purposes to NCDs treatment [5]. In 2012, the Ministry of Health and Medical Education (MOHME) of Iran established the Iranian NCD committee (INCDC) as the leadership political action committee aiming to implement the national prevention and monitoring framework [6].

In 2013, the INCDC funded a well-designed nationwide cohort study (Prospective Epidemiological Research Studies in IrAN or PERSIAN) aiming to investigate the burden and risk factors of NCDs in different ethnic groups distributed in 18 distinct geographic regions of Iran [7]. The PERSIAN cohort study is aimed to underscore the importance of prioritizing the country’s action to reduce insufficient physical activity, sodium intake and hypertension, substance abuse rate, halt the rise of obesity and diabetes, and improve the coverage of treatment for prevention of cardiovascular disorders [8]. A total of 180,000 individuals aged 35–70 years will be enrolled and followed for 15 years after registration. Seventeen universities of medical sciences are responsible for filling the questionnaires and carrying out the minimal predefined process determined by the central committee in INCDC [7].

Mashhad, as the second largest city of Iran, has been selected to include 12,000 personnel of the Mashhad University of Medical Sciences representing the northeastern population. All PERSIAN cohort sites are meant to include representative samples of the small city population. Mashhad is the first site located in the metropolitan area which significantly differs with regards to the pathogenic and salutogenic health determinants (e.g. migration rate, air pollution, lifestyle, cultural and economic issues, education, entertainment facilities, etc.) [9–11]. We herein describe the protocol and the baseline assessment of the PERSIAN Organizational Cohort study in Mashhad University of Medical Sciences (POCM).

## Method and design

### Target population

The POCM was designed to prospectively recruit 12,000 of 15,019 current personnel of the Mashhad University of Medical Sciences (MUMS). The organization consists of 35 sub-sections including, 14 hospitals, 7 faculties, 7 vice chancelleries, 5 health centers, 3 research institution, and the headquarter department. Besides, a total of 53 emergency centers are distributed around the city. Among 12,865 eligible personnel, about 5.9% were faculty staff in 84 academic departments and 75.4% of the staffs were directly involved in the treatment process. The mean age of personnel was 39.4 ± 8.9 years of which 44.4% are male. It should also be noted that about 69.3% of the target population had an academic degree. The eligibility criteria were designed to maximize the recruitment of the community-dwelling middle-aged adults and minimize loss to follow up. Individuals will be eligible for inclusion if they are aged between 30 and 70 years at the time of enrolment, are of Iranian descent, are living in Mashhad for at least 9 months of the year, and have no plans to move within the next 2 years. The persons who are not able to articulate their own opinions due to physical or intellectual disabilities are excluded from the study.

Thus far, a total of 5287 adults have been enrolled during the first 19 months. Table 1 displays the baseline characteristics and descriptive statistics of the variables collected through face-to-face interviews. Table 2 shows psychological status of the included individuals and Table 3 abstracts the data collected using standard tests/examinations.

**Table 1:** Characteristics of the included participants collected using face-to-face interview

Variable	Total (*n* = 5287)	Male (*n* = 2427)	Female (*n* = 2860)	***P***-value^**a**^
**Demographic**				
**Age** (year)	43.9 ± 7.6	45.2 ± 8.1	42.8 ± 7.0	**< 0.001**
**Ethnicity**				
Fars	4486 (84.8%)	2060 (84.9%)	2426 (84.8%)	0.881
Turk	241 (4.6%)	114 (4.7%)	127 (4.4%)	
Kurd	194 (3.7%)	91 (3.7%)	103 (3.6%)	
Others	366 (6.9%)	162 (6.7%)	204 (7.1%)	
**Socioeconomic**				
**Education Level**				
Illiterate	7 (0.1%)	4 (0.2%)	3 (0.1%)	**< 0.001**
Elementary	88 (1.7%)	49 (2%)	39 (1.4%)	
Diploma	734 (13.9%)	434 (17.9%)	300 (10.5%)	
Short-cycle tertiary education	483 (9.1%)	206 (8.5%)	277 (9.7%)	
Bachelor	2339 (44.2%)	802 (33%)	1537 (53.7%)	
Master	883 (16.7%)	403 (16.6%)	480 (16.8%)	
PhD or higher	753 (14.2%)	529 (21.8%)	224 (7.8%)	
**Marital Status**				
Single	447 (8.5%)	60 (2.5%)	387 (13.5%)	**< 0.001**
Married	4584 (86.7%)	2340 (96.4%)	2244 (78.5%)	
Widowed	70 (1.3%)	6 (0.2%)	64 (2.2%)	
Divorced	175 (3.3%)	19 (0.8%)	156 (5.5%)	
Other	11 (0.2%)	2 (0.1%)	9 (0.3%)	
**Job Category**				
Nurses and midwives	976 (18.5%)	198 (8.2%)	778 (27.2%)	**< 0.001**
Administrative and office personnel	825 (15.6%)	371 (15.3%)	454 (15.9%)	
Faculty Staff	505 (9.6%)	387 (15.9%)	118 (4.1%)	
Non-nursing health care assistants	468 (8.9%)	172 (7.1%)	296 (10.3%)	
Technicians and assistants in health	276 (5.2%)	100 (4.1%)	176 (6.2%)	
Biomedical and Medical Specialists	163 (3.1%)	78 (3.2%)	85 (3%)	
Other	2074 (39.2%)	1121 (46.2%)	953 (33.3%)	
**House Ownership**				
Private	4094 (77.4%)	1899 (78.3%)	2195 (76.8%)	0.322
Rented	1001 (18.9%)	445 (18.3%)	556 (19.4%)	
Organizational	21 (0.4%)	12 (0.5%)	9 (0.3%)	
Borrowed	171 (3.2%)	71 (2.9%)	100 (3.5%)	
**Life Style**				
**Physical Activity**				
Aerobic Exercise (min)	13 ± 22.4	16 ± 25.6	11 ± 18.9	**< 0.001**
Heavy Exercise (min)	1 ± 7.8	2 ± 10.5	0.6 ± 4.3	**< 0.001**
Desk Work (min)	73 ± 90.3	87 ± 98.1	62 ± 81.3	**< 0.001**
Computer Work (min)	182 ± 142.8	180 ± 146	184 ± 140	0.275
TV Watching (min)	88 ± 60.5	95 ± 59.6	81 ± 60.5	**< 0.001**
Mobile Use (min per day)	71.2 ± 76.8	67.9 ± 75.3	74 ± 77.9	**0.004**
**Sleep Assessment**				
Night Sleep Duration (min)	382 ± 64.9	382 ± 64.3	383 ± 65.3	0.704
Has Daytime Sleep	3660 (69.2%)	1607 (66.2%)	2053 (71.8%)	**< 0.001**
Has Night Working Shift	1724 (32.6%)	903 (37.2%)	821 (28.7%)	**< 0.001**
**Clinical History**				
Diabetes Mellitus	258 (4.9%)	158 (6.5%)	100 (3.5%)	**< 0.001**
Hypertension	463 (8.8%)	250 (10.3%)	213 (7.4%)	**< 0.001**
Ischemic Heart Diseases	175 (3.3%)	92 (3.8%)	83 (2.9%)	0.076
Myocardial Infarction	32 (0.6%)	28 (1.2%)	4 (0.1%)	**< 0.001**
Stroke	19 (0.4%)	15 (0.6%)	4 (0.1%)	**0.005**
Rheumatism	109 (2.1%)	32 (1.3%)	77 (2.7%)	**< 0.001**
Epilepsy	15 (0.3%)	8 (0.3%)	7 (0.2%)	0.611
Multiple Sclerosis	15 (0.3%)	3 (0.1%)	12 (0.4%)	0.066
Colorectal Cancer	4 (0.1%)	4 (0.2%)	0 (0%)	**0.044**
Osteoporosis	226 (4.3%)	31 (1.3%)	195 (6.8%)	**< 0.001**
Fatty Liver	814 (15.4%)	467 (19.2%)	347 (12.1%)	**< 0.001**
Renal Failure	15 (0.3%)	6 (0.2%)	9 (0.3%)	0.797
Thyroid Disorders	594 (11.2%)	104 (4.3%)	490 (17.1%)	**< 0.001**
Alopecia	2784 (52.7%)	1645 (67.8%)	1139 (39.8%)	**< 0.001**
**Substance Abuse**				
Smoking				
Current Smoker	253 (4.8%)	235 (9.7%)	18 (0.6%)	0.122
Ex-Smoker	404 (7.6%)	381 (15.7%)	23 (0.8%)	**< 0.001**
Hookah	507 (9.6%)	365 (15%)	142 (5%)	**< 0.001**
Alcohol	112 (2.1%)	94 (3.9%)	18 (0.6%)	**< 0.001**
Drug Abuse	59 (1.1%)	55 (2.3%)	4 (0.1%)	**< 0.001**
**Gynecology**				
Menstruation Age (year)	NA	NA	13.4 ± 3.8	NA
Menopause	NA	NA	411 (14.4%)	NA
Pregnancy Number	NA	NA	2.2 ± 1.5	NA
Has Death Birth Child	NA	NA	91 (3.2%)	NA
Ovary Removal	NA	NA	57 (2%)	NA
Hysterectomy	NA	NA	94 (3.3%)	NA
Infertility	NA	NA	164 (5.7%)	NA
Breast Cancer	NA	NA	19 (0.7%)	NA
Ovarian Cancer	NA	NA	1 (0.03%)	NA
**Food Frequency** (kcal)				
Bread and cereals	876 ± 695	1051 ± 847	728 ± 486	**< 0.001**
Meat and Related Products	272 ± 194	306 ± 173	243 ± 205	**< 0.001**
Milk and Dairy	409 ± 250	417 ± 240	402 ± 259	**0.033**
Vegetables	266 ± 156	247 ± 148	281 ± 160	**< 0.001**
Oil, oilseeds, and butter	466 ± 299	470 ± 298	462 ± 300	0.376
Sugars	111 ± 172	138 ± 131	87 ± 197	**< 0.001**
Total Calorie Intake	3079 ± 1252	3364 ± 1392	2837 ± 1061	**< 0.001**
Water (glass per day)	2.9 ± 2.5	2.9 ± 2.4	2.9 ± 2.6	0.655

Notes: Values are presented as mean ± SD or N (%)

^a^Analysis by independent-samples T test, Fisher’s exact test (2-sided), or Chi-square test

*Abbreviations*: *DASS-21* Distress, Anxiety, and Stress Scale, *HSS-35* Hospital Stress Scale

**Table 2:** Self-reported level of stress, anxiety, and depression in study population collected using the DASS-21 questionnaire

Variable	Total (*n* = 3353)	Male (*n* = 1545)	Female (*n* = 1808)	***P***-value ^**a**^
**Depression (0–42)**				
Normal	1578 (47.1%)	788 (51%)	790 (43.7%)	< 0.001
Mild	696 (20.8%)	311 (20.1%)	385 (21.3%)	
Moderate	702 (20.9%)	302 (19.5%)	400 (22.1%)	
Severe	271 (8.1%)	107 (6.9%)	164 (9.1%)	
Extremely Severe	106 (3.2%)	37 (2.4%)	69 (3.8%)	
**Anxiety (0–42)**				
Normal	1200 (35.8%)	600 (38.8%)	600 (33.2%)	< 0.001
Mild	360 (10.7%)	162 (10.5%)	198 (11%)	
Moderate	750 (22.4%)	345 (22.3%)	405 (22.4%)	
Severe	491 (14.6%)	225 (14.6%)	266 (14.7%)	
Extremely Severe	552 (16.5%)	213 (13.8%)	339 (18.8%)	
**Stress (0–42)**				
Normal	2771 (82.6%)	1315 (85.1%)	1456 (80.5%)	0.001
Mild	276 (8.2%)	117 (7.6%)	159 (8.8%)	
Moderate	217 (6.5%)	89 (5.8%)	128 (7.1%)	
Severe	78 (2.3%)	21 (1.4%)	57 (3.2%)	
Extremely Severe	11 (0.3%)	3 (0.2%)	8 (0.4%)	

Notes: Values are presented as mean ± SD or N (%)

^a^Analysis by independent-samples T test or Chi-square test

**Table 3:** Clinical measurements collected using standard tests and examinations

Variable	Total (*n* = 5287)	Male (*n* = 2427)	Female (*n* = 2860)	***P***-value^**a**^
**Anthropometry**				
Height (cm)	165.2 ± 9.8	172.8 ± 7.7	158.7 ± 6.1	**< 0.001**
Weight (kg)	73.7 ± 13.4	80.3 ± 12.6	68 ± 11.4	**< 0.001**
BMI (kg/m^2^)	27 ± 5.26	26.97 ± 6.24	27.01 ± 4.25	0.82
Abdomen Circumference (cm)	93 ± 10.7	95.6 ± 10.8	90.8 ± 10	**< 0.001**
Neck Circumference (cm)	37 ± 4.1	38.7 ± 4.4	35.5 ± 3.1	**< 0.001**
**Bio-impedance**				
Total Body Water (liter)	35.8 ± 7.8	42.3 ± 5.9	30.2 ± 3.9	**< 0.001**
Body Fat Mass (kg)	25 ± 8.1	22.7 ± 7.7	27 ± 7.9	**< 0.001**
Protein (kg)	9.6 ± 2.1	11.4 ± 1.6	8 ± 1.1	**< 0.001**
Mineral (kg)	3.3 ± 0.9	3.9 ± 1	2.9 ± 0.4	**< 0.001**
**Laboratory**				
Hyperglycaemia (FBS > 125 mg/dL)	265 (5.1%)	170 (7%)	95 (3.4%)	**< 0.001**
Hypercholesterolemia (Chol> 240 mg/dL)	305 (5.8%)	146 (6%)	159 (5.6%)	0.555
Hypertriglyceridemia (TG > 150 mg/dL)	1257 (24%)	832 (34.3%)	425 (15.1%)	**< 0.001**
Low HDL (HDL < 40 mg/dL)	330 (6.3%)	252 (10.4%)	78 (2.8%)	**< 0.001**
**Pulse-Wave Analysis**				
Central Systolic Pressure (mmHg)	108 ± 12	110 ± 12	105 ± 11.4	**< 0.001**
Central Diastolic Pressure (mmHg)	74 ± 8.9	76 ± 9.1	71 ± 8.1	**< 0.001**
Central Mean Pressure (mmHg)	87 ± 10.1	89 ± 10.3	85 ± 9.4	**< 0.001**
Pulse-Wave Velocity (m/s)	7.2 ± 1.6	7.6 ± 1.6	6.7 ± 1.5	**< 0.001**
Central Pulse Pressure (mmHg)	34 ± 6.8	34.4 ± 6.9	33.5 ± 6.8	**0.002**
Augmentation Index (AIx) (%)	26.7 ± 11.5	23.5 ± 10.9	29.7 ± 11.2	**< 0.001**
**Electrocardiography**				
Heart Rate (bpm)	69 ± 14.7	67 ± 10.3	71 ± 17.7	**< 0.001**
Bradycardia	693 (13.1%)	450 (18.5%)	243 (8.5%)	**< 0.001**
Tachycardia	32 (0.6%)	20 (0.8%)	12 (0.4%)	0.178
Heart Axis				
Normal (−30° to + 90°)	5007 (94.7%)	2244 (92.5%)	2763 (96.6%)	**< 0.001**
Left Axis Deviation (−30° to −90°)	164 (3.1%)	117 (4.8%)	47 (1.6%)	
vRight Axis Deviation (+ 90° to 180°)	100 (1.9%)	57 (2.3%)	43 (1.5%)	
Indeterminate Axis(−90° to 180°)	16 (0.3%)	9 (0.4%)	7 (0.2%)	
PR Interval (ms)	148 ± 31.2	152 ± 38.9	143 ± 20.7	**< 0.001**
QRS Interval (ms)	93 ± 20.8	97 ± 25.4	90 ± 14.8	**< 0.001**
T Interval (ms)	196 ± 50.6	195 ± 47.6	198 ± 53.3	0.082
ST Interval (ms)	104 ± 14.7	100 ± 15.9	107 ± 12.5	**< 0.001**
QT (ms)	396 ± 28.1	393 ± 28.1	398 ± 27.9	**< 0.001**
QTcB (ms)	424 ± 104.1	416 ± 106.8	431 ± 101.1	**< 0.001**
QTcF (ms)	415 ± 91.7	409 ± 91.9	420 ± 91.2	**< 0.001**
**Spirometry**^**b**^				
FEV1/FVC (%)	82.7 ± 7.6	82.1 ± 7.36	83.1 ± 7.72	**< 0.001**
Normal (> = 70%)	4497 (95.6%)	2058 (95.3%)	2439 (95.8%)	0.602
Mild Obstruction (60–69%)	158 (3.4%)	79 (3.7%)	79 (3.1%)	
Moderate Obstruction (50–59%)	37 (0.8%)	15 (0.7%)	22 (0.9%)	
Severe Obstruction (< 50%)	13 (0.3%)	7 (0.3%)	6 (0.2%)	
**Orthopedic**^**c**^				
Right Pinch Strength (lbs)	10 ± 4.8	13.7 ± 4	7 ± 3.1	**< 0.001**
Left Pinch Strength (lbs)	9.8 ± 4.7	13.3 ± 4	7 ± 3	**< 0.001**
Right Grip Strength (lbs)	54.1 ± 24.1	73.6 ± 19.5	38.1 ± 13.3	**< 0.001**
Left Grip Strength (lbs)	51 ± 23.5	69.5 ± 19.7	35.9 ± 13.2	**< 0.001**
Carpal tunnel syndrome	264 (5%)	44 (1.8%)	220 (7.7%)	**< 0.001**
Calcaneal Spur	26 (0.5%)	7 (0.3%)	19 (0.7%)	0.059
Knee Arthritis	48 (0.9%)	8 (0.3%)	40 (1.4%)	**< 0.001**
**Smell-Taste Assessment** (*N* = 586)^d^				
Smell Score (0–6)	5.1 ± 1.3	4.9 ± 1.5	5.3 ± 1.1	**< 0.001**
Taste Score (0–12)	9 ± 2.7	8.1 ± 2.9	9.9 ± 2.2	**< 0.001**
**Ultrasound**				
**Abdominopelvic**				
Right Kidney Width (mm)	106 ± 8.6	107 ± 8.6	104 ± 8.4	**< 0.001**
Left Kidney Width (mm)	107 ± 9.4	109 ± 7.9	105 ± 10.1	**< 0.001**
Spleen Width (mm)	102 ± 13.9	107 ± 14.3	98 ± 12.5	**< 0.001**
SAT (mm)	21.7 ± 8.4	22.3 ± 8.9	21.2 ± 7.9	**0.041**
VAT (mm)	74 ± 22.6	82 ± 22.6	67 ± 20.3	**< 0.001**
Liver Craniocaudal Diameter (mm)	101 ± 12.2	106 ± 12	97 ± 10.6	**< 0.001**
Renal Aorta Diameter (mm)	14.5 ± 4.7	15.3 ± 2.1	13.7 ± 6	**< 0.001**
**Thyroid**				
Right Lobe Volume (cc)	5.51 ± 3.56	6.09 ± 3.43	5 ± 3.58	**< 0.001**
Left Lobe Volume (cc)	5.86 ± 3.88	6.71 ± 4.09	5.12 ± 3.54	**< 0.001**
Isthmus Volume (cc)	0.66 ± 0.87	0.78 ± 1.14	0.55 ± 0.51	**< 0.001**
One Nodule	1464 (27.7%)	572 (23.6%)	892 (31.2%)	**< 0.001**
Two Nodules	550 (10.4%)	204 (8.4%)	346 (12.1%)	**0.001**
Three Nodules	259 (4.9%)	77 (3.2%)	182 (6.4%)	**< 0.001**
Four Nodules	106 (2%)	28 (1.2%)	78 (2.7%)	**0.001**
Five Nodules	42 (0.8%)	12 (0.5%)	30 (1%)	0.085

Notes: Values are presented as mean ± SD or N (%)

^a^Analysis by independent-samples T test, Fisher’s exact test (2-sided), or Chi-square test

^b^Patients who had at least one contraindication for Spirometry were excluded

^c^The frequency of disorders are the sum of left and right hands/foots

^d^The smell-taste test was performed only for the high-risk subpopulation

*Abbreviations*: *BMI* Body Mass Index, *FBS* Fasting Blood Sugar, *Chol* Cholesterol, *TG* Triglycerides, *HDL* High-Density Lipoproteins, *TSH* Thyroid Stimulating Hormone, *PSA* Prostate-Specific Antigen, *FEV1/FVC* Forced Expiratory Volume/Forced Vital Capacity, *SAT* Subcutaneous Adipose Tissue, *VAT* Visceral Adipose Tissue, *NA* Not Applicable

### Human resources

An integrated team consisting of 16 members (three general interviewers, three nutrition interviewers, three nurses, three sampling technicians, an orthopedic examiner, a radiologist, a general physician, and a field supervisor) is responsible for consistent data collection. All team members were required to complete two rounds of 3-day training workshops in the process of interviewing or performing medical examinations. A 3-month gap between two workshops was considered as the pilot period to minimize the questioning errors. At the end of the second workshop, final team members were selected by theoretical and practical exams administered by the central committee in INCDC. The field supervisor is in charge of surveillance of data completeness assuring that the process is carried out according to the protocols. A driver is also responsible for the transportation of biological samples to the central biobank building.

A scientific committee of Co-Principal Investigators (Co-PIs) including pharmacologists, cardiologists, radiologists, urologists, ophthalmologists, orthopedists, nutritionists, pulmonologists, epidemiologists, and occupational therapists performs and audits the scientific aspect of the data gathering process. They also observe that the protocols and proposals related to the subprojects are being performed in the POCM framework. The main PI is the corresponding manager and the lead researcher who ultimately verifies the administrative issues.

### Office

The enrollment and data collection process are conducted in a 450 m^2^ (30 m × 15 m) office located in Emam Reza Hospital as the largest academic hospital in Mashhad. The office consists of one reception desk and 21 rooms, including seven for interviews, eight for examinations, two for follow-ups, sampling and laboratory, research room, and conference. All facilities and devices required for examinations and sampling are provided in the office.

### Ethics approval and consent to participate

The study protocol follows the principles laid down in the Helsinki declaration and has been approved by the ethics committee of the Iranian MOHME, and the institutional review board of the MUMS (IR.MUMS.REC.1395.526; January 2017). The informed consent is obtained from all participants prior to the baseline survey and they can make an informed decision about whether or not to continue participation at any time. The findings of the study will be disseminated in peer-reviewed journals, appropriate national or international conferences, and specialist interest groups in scientific social media. All data are stored in a codified database with automatic backup schedule at regular intervals.

### Enrollment process

**Fig. 1:**
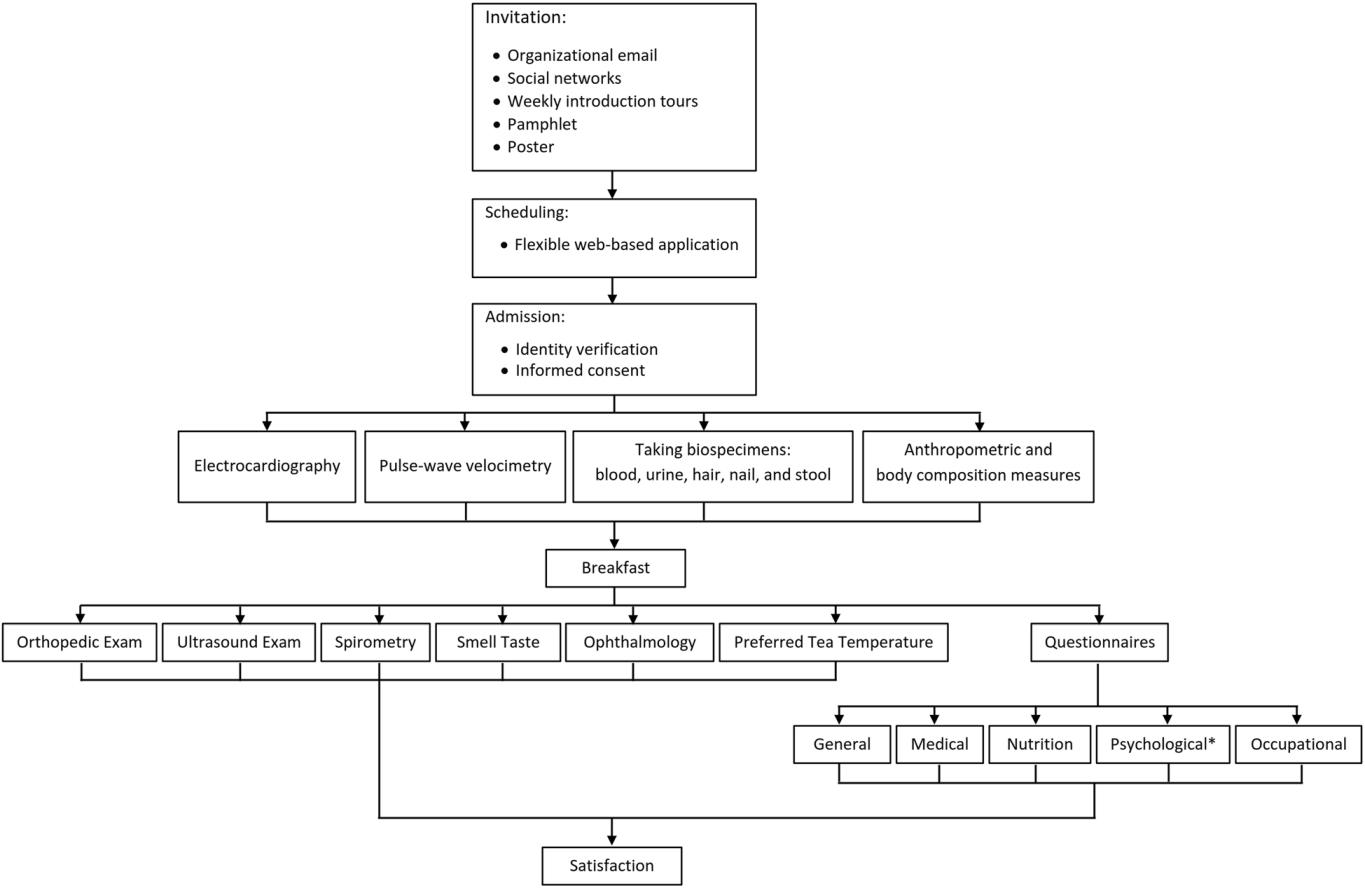
The schematic presentation of the enrollment process of the Mashhad’s PERSIAN Organizational Cohort Study. * Psychological questionnaires are self-administered and include DASS-21, PSQI, and Steinmetz or HSS-35 job stress questionnaires

A schematic presentation of the enrollment process is presented in Fig. 1. Below we explain each step in detail.

#### Invitation and coordination

An invitation email containing a brief explanation of the study and enrollment process, inclusion and exclusion criteria, a link to the online scheduling system, office’s phone numbers, and address is sent to the organizational email address of the personnel working in each sub-organization. Three prerequisite points including 10–12 h fasting, hair-nail sampling and specific test requirements, and necessary identification documents are also noted in the email content. Other invitation methods include advertisement in social media, weekly 1-h introduction tours for faculty staff, distributing pamphlets, and installing posters on advertisement boards of sub-organizations. Once a volunteer reserves a specific date via the scheduling system, a short message containing the date and aforementioned prerequisite points is sent to her/his cellphone. Also, the registrant is responsible for making a phone call for final reminding and coordination 1 day before the scheduled date.

#### Registration

The registrant checks the personal identification cards to ensure that participants meet all inclusion criteria upon arrival. The written informed consent is obtained, and an 11-digit unique identifier (PCID) is assigned to each participant, which is used to label all biological samples and documentations.

#### Laboratory and biobank

The participants are asked to maintain fasting state for 10–12 h and not to colour or trim their hair and nail for at least 2 weeks. About 25 ml of blood is taken using one 9 ml clot and three 6 ml EDTA Vacutainer (Greiner Bio-One International GmbH, Kremsmunster, Austria). The blood is centrifuged and fractioned into fifteen 1 ml CryoTubes in 96–1 Racks (Micronic, Lelystad, the Netherlands) for each participant and are stored in − 80 °C freezers (i.e. 3 whole bloods, 7 plasmas, 3 buffy coats, and 2 serums). Urine is also stored in two 1.5 ml tubes in − 20 °C freezers. About 200 strands of hair (1–3 cm) and all fingernails and toenails are stored in foil and zip-lock bags with humidity absorber in cool dry rooms. Since the central biobank building is 6.3 km away from the office, the blood and urine samples are stored temporarily in − 20 °C freezers and are transported to the permanent freezers twice a week. The central biobank is equipped with four − 80 °C freezers (GFR Ultra-low Freezer with CO_2_ Backup-System, Lübeck, Germany), two − 20 °C freezers (ARCTIKO, Esbjerg, Denmark), a 17 m^2^ cold room, uninterruptible power supply system, dehumidifier, and real time temperature controller.

The cell blood count test (NK Alpha, Nihon Kohden, Tokyo, Japan) and the following biochemistry tests (BT1500 auto analyzer, Biotecnica Instruments, Rome, Italy) are also performed: concentrations of fasting blood sugar, blood urea nitrogen, creatinine, liver function indices (i.e. alanine transaminase, aspartate transaminase, alkaline phosphatase, and γ-glutamyl transpeptidase), and lipid profile (i.e. total cholesterol, high density lipoprotein cholesterol, and triglycerides). The urine analysis includes the assessment of urine pH, specific gravity, the presence of glucose, blood, protein, nitrates, ketones, bilirubin, albumin, ascorbic acid, and leukocytes. The Total Prostate-Specific Antigen (TPSA) level is measured (MINI VIDAS, Biomerieux, Lyon, France) for the men aged 45 years or older to screen for prostate cancer. The Thyroid Stimulating Hormone (TSH) is also measured for all participants aiming to make an initial screening of hypo- and hyperthyroidism [12].

#### Fecal Occult Blood (FOB) test

Since colon cancer is one of the most prevalent and the second lethal malignancy in both men and women around the world, early detection is imperative [13]. The FOB test is a noninvasive, rapid, and easy-to-carry diagnostic test which uses antibodies to discern occult blood (macroscopically invisible) in the stool. It has been shown that using the FOB test as a screening tool results in decreased morbidity and mortality due to colon cancer [14]. In POCM the FOB test is performed for the high risk subpopulation who are older than 50 years of age, have a (family) history of colon cancer, polyp, colitis, and self-reported intestinal disorders. Once the participant is unwilling or unable to provide the stool sample in the center, a full oral explanation about the procedure (with pamphlet) is given and he/she is asked to perform the test at home. If the FOB result is positive or the participant reports the history of visible blood in stool, he/she is referred for colonoscopy.

#### Anthropometry and body composition

Weight (in kg), height (in cm), and body circumferences (i.e. neck, waist, hip, and wrist in cm) are measured to the nearest one decimal point using US National Institutes of Health protocols [15]. The bioelectrical impedance analysis is also performed using Inbody 770 (Inbody Corporation, Seoul, Korea). The machine sends a weak alternating current (50–1000 kHz) which is resisted by body tissues. The individuals are required to step on the footplate barefoot, hold the hand electrodes, and keep the arms straight while not touching the thighs and armpits area. Body composition measurements include intracellular and extracellular water, total body water, skeletal muscle mass, visceral fat level and area, bone mineral content, body cell mass, basal metabolic rate, whole and segmental phase angel, waist-hip ratio, obesity degree, and weight control indices. To increase the accuracy of the measures, main indices are also reported for each specific five body cylinders (i.e. right arm, left arm, trunk, right leg, and left leg).

#### Measurement of sitting height

Previous investigations have explored different associations between the sitting height and chronic disease mortality. A recent paper from the extensive European Prospective Investigation into Cancer and Nutrition (EPIC) study comprising more than 409,000 individuals found that sitting height was inversely associated with the mortality of the respiratory and circulatory disease [16]. Since there is a paucity of data describing this relationship among Asian ethnicities, the sitting height is measured in the POCM using the standard procedure [17]. The participant is asked to put the thighs closely together on the anthropometric chair such that a right angle is formed between the thighs and the trunk. While the shoulder blades, back of the head, and buttocks of the subject touch the vertical board, the one decimal point distance between the sitting place and the highest point of the head is measured as sitting height.

#### Cardiovascular evaluation

The Central Blood Pressure (CBP) and Pulse Wave Velocity (PWV) are measured using the SphygmoCor XCEL System (AtCor Medical Incorporation, Sydney, Australia), which is a non-invasive diagnostic tool for evaluating the arterial stiffness. The test requires at least 6 h fasting state and no alcohol, tobacco, and caffeine use for 12 h. The test is performed after 15 min rest in the supine position. In CBP analysis, the system derives the central aortic pressure waveform from cuff pulsations recorded at the brachial artery. Analysis of the waveform results in key parameters including central systolic pressure, central pulse pressure, and indices of arterial stiffness. In PWV analysis, the velocity of the arterial pulse waveform travelling through the descending aorta to the femoral artery is measured. A tonometer is used to detect the carotid pulse while the femoral pulse is measured through pulsations in a cuff placed around the thigh. Higher CBP and PWA indices have been shown to be markers of cardiovascular risk [18–20].

Dual-arm Blood Pressure (BP) is measured after 2–3 min rest in the seated position. A cuff tailored to their individual mid-arm circumference is used and a trained nurse conducts BP measurements in accordance with the standardized auscultatory method protocol [21] using an analog sphygmomanometer (Sanaphon, Rudolf Riester GmbH, Brückstr, Germany). Systolic and diastolic blood pressures in addition to pulse rate are measured two times at a 10-min interval.

A 12-lead electrocardiogram is recorded after 5-min rest in the supine position (Touch ECG HD+, Technomed Cardiology, Marden, United Kingdom). Each day, a trained nurse codifies the results according to the Minnesota Coding system [22]. The quality of coded results is controlled by random sampling by the cardiologists.

#### Orthopedic examination

A total of 13 self-report questions and 15 examinations are used to evaluate the following disorders: carpal tunnel syndrome, lateral epicondylitis, De Quervain’s tendinitis, trigger finger, ganglion cyst, Dupuytren’s contracture, first carpometacarpal arthritis, frozen shoulder, impingement syndrome, cervical and lumbar discopathy, knee arthritis, and calcaneal spur. Grip and pinch strengths of both hands are measured using hand dynamometers (Lafayette Instrument Company, Indiana, United States). The 3-dimension images of hands and feet are also scanned (Payafanavaran Company, Mashhad, Iran) and stored for future measurements and analyses. In addition, a footprint scanner is used to save the image representing the distribution of pressure on feet, while the person is making a regular 3-step walk on a 5-m walkway.

#### Abdominopelvic ultrasound examination

A subgroup of participants undergoes a standardized ultrasound examination using a high-end ultrasound equipment (Affiniti 50G, Philips, Germany). Routine abdominal ultrasound examination is performed aiming to fulfill the prepared structured form. In addition, careful examination of the liver is performed to investigate the prevalence of Non-Alcoholic Fatty Liver Disease (NAFLD). First, the whole visible liver parenchyma is examined using a 3–5 MHz convex transducer. Liver parenchyma is examined by lateral and intercostal approaches in the axial and longitudinal planes. The presence and grading of NAFLD are recorded as follows: 1) mild (slightly increased echogenicity with normal visualization of the diaphragm and the intrahepatic vessels), 2) moderate (moderately increased echogenicity with slightly impaired visualization of the diaphragm or intrahepatic vessels), and 3) severe (markedly increased echogenicity with poor or no visualization of the diaphragm or intrahepatic vessels) [23]. The liver is assessed to be normal if normal liver parenchyma with homogeneous echotexture is detected. The presence of any cystic or solid hepatic mass is also recorded. Gallbladder is evaluated for the presence of any congenital abnormality, gallbladder stones, or sludge. Biliary ducts are evaluated for the presence of dilatation or intraductal lesion. Spleen is examined using a left intercostal approach, and its largest diameter and anteroposterior diameter are recorded. The presence of any parenchymal lesion is also recorded. Both kidneys are examined and their dimension, cortical thickness, echotexture, and presence of renal stones are recorded.

#### Breast Cancer screening

The high risk subpopulation (women older than 45 years old) is referred to mammography (MicroDose SI Mammography Machine, Philips, Germany). A radiologist inspects the Mediolateral-Oblique (MLO) and Cranial-Caudal (CC) views on a medical monitor (MDCC-6430, Barco, Belgium) to detect any mass or calcifications as well as architectural distortion, nipple retraction, axillary adenopathy, skin retraction, skin thickening, and trabecular thickening. The following variables are determined for each specific mass: shape, size, margin, laterality, location. The marked image of the suspected area is also recorded. The final evaluation is scored according Breast Imaging Reporting and Data System (BI-RADS). Supplementary ultrasound screening is also performed when needed by the same radiologist to make final diagnosis and provide clinical recommendations.

#### Thyroid ultrasound examination

Thyroid ultrasonography is performed using a linear 7.5–12.5 MHz transducer in supine position with neck hyperextension. The size of thyroid lobes as well as the isthmus is measured as maximum diameter on the basis of longitudinal, anteroposterior, and transverse measurements in centimeters. The volume of each lobe is calculated by multiplying three dimensions in 0.52. Total thyroid volume is determined by adding up the volume of isthmus to left and right lobes. Echogenicity of the thyroid was categorized as homogenous and heterogeneous. Hypo-echogenicity was defined as thyroid echogenicity less than echogenicity of strap muscles [24]. Any space occupying lesion in the thyroid is evaluated for composition, echogenicity, size, shape, margin and echogenic foci according to the ACR lexicon and TIRADS score is determined for each nodule [25]. Up to 5 largest nodules are recorded for each patient. FNA from the suspicious thyroid nodule is suggested for the patients according to the size of the nodule and ATA guideline [26].

#### Ophthalmology examination

Uncorrected and best spectacle corrected visual acuity are measured for all participants using Snellen chart display (TOOSNEGAH, Razavi Khorasan, Iran). Automated, subjective and cyclo-refraction are performed using an auto kerato-refractometer machine (KR-1, TOPCON, Tokyo, Japan). The non-contact tonometry and pachymetry is also performed using the CT-1P (TOPCON, Tokyo, Japan). The CT-1P also calculates the adjusted Intra Ocular Pressure (IOP) using the measured corneal thickness which is known as the corrected IOP. The Photo Slit Lamp LS6 (KMED, Chungking, China) is used to record a photography of a cornea, iris and lens with and without pupillary dilation. First, a diffuse beam used with low magnification to take anterior segment photography. Then, the light-beam width and height are fixed at 8 and 2 mm, respectively and the slit beam is locked at an angle of 60 degrees for corneal slit-photography. Retro-illumination photography is taken after pupillary dilation. The Optical Coherence Tomography Angiography (OCTA) is also performed using the Optovue system (Optovue, California, United States) to analysis optic nerve head, nerve fiber layer, macula, retinal vasculature, foveal avascular zone, and image artifacts. An auto-focus and auto-capture non-mydriatic retinal camera (TRC-NW400, TOPCON, Tokyo, Japan) was also used to obtain high resolution color images of the retina and the anterior segment of the eye. The examinations are performed by two trained optometrists and the results are interpreted by the ophthalmologists using a remote monitoring system.

#### Spirometry

A trained nurse performs Spirometry test to assess the participants’ pulmonary Forced Vital Capacity (FVC), using Spirobank spirometer (MIR, Rome, Italy) that has been previously proven to be reliable for research purposes [27]. The machine is connected to a computer using the Winspiro Pro software that provides real-time feedback on the flow-volume curves, repeatability of the test, and a quality indicator (A to F). According to the American Thoracic Society/European Respiratory Society (ATS/ERS) standards, the FVC maneuver is performed as follows: 1) maximal inhalation, 2) a blast of exhalation, and 3) continued exhalation to the end of the test [28]. Once the participant has at least one of the following contraindications, he/she is excluded: recent heart surgery, eye surgery, cardiac arrest, stroke, myocardial infarction, laparotomy, pneumothorax, tuberculosis, and current systolic blood pressure higher than 200 mmHg, or diastolic blood pressure higher than 140 mmHg [29].

#### Smell-taste function evaluation

It is estimated that about 14 and 17% of adults exhibit functional olfactory and gustatory impairments [30]. It has been shown that the following risk factors are likely to affect the smell-taste function: having 50 years of age or older, neurological disorders (i.e. multiple sclerosis, seizure, Parkinson, central nervous system tumors, history of cerebrovascular accident, cognitive impairments, head trauma, dementia, Alzheimer, and etc.), respiratory disorders (i.e. asthma, allergy, polyp, infections, and etc.), substance abuse (i.e. alcohol, cigarettes, hookah, and etc.), medication intake (i.e. anti-diabetics, anti-hypertensive, corticosteroids, vasodilators, anti-rheumatic, and etc.), and other reasons (e.g. dentures, hypothyroidism, liver diseases, postmenopause, and etc.). In the POCM, the smell function is quantified using a standard six multiple-choice questions in which the participant is asked to scratch the smell-covered area and choose the correct answer. The score ranges between 0 and 6, where a higher score represents better smell function. The gustatory system is evaluated using 12 tasted spoons in four tastes (i.e. sweet, sour, salty, and bitter) and three levels (low, moderate, and high). The taste score is calculated assigning one point to each correct answer, which results in a minimum and maximum score of 0 and 12, respectively. A random number of individuals with none of the mentioned risk factors are also subjected to the smell-taste evaluation as control group.

##### Tea temperature measurement

Since previously published papers have reported contradictory findings on the association between drinking hot beverages and esophageal squamous cell carcinoma [31–33], collecting precisely objective data will help future researchers to assess this relationship explicitly. Since black tea is the most commonly used hot beverage in Iran, we measured the preferred black tea temperature objectively using a digital liquid thermometer TP500 (range: -50 °C ~ 350 °C; error: ±1 °C; Guangdong, China). We used the method which has been previously proposed and validated by Pourshams et al. [34]. Briefly, two fresh cups of tea are poured for the staff and participant. The participant is asked to sip the tea at 75 °C and repeatedly at regular 2.5 °C lower temperatures. Once the participant states that the tea is at her/his usual favorable temperature, the process is terminated and the temperature of the participant’s cup is recorded. The decremental process continues at most until 50 °C. The temperature for the participants who prefer lower temperatures is recorded as < 50 °C. The following related factors are also recorded: self-reported number of 250 cc cups of black, green, and herbal tea per day/month/year, self-reported time between tea pouring and drinking, steep duration, and start/quit age of tea drinking.

#### Questionnaires

A total of 508 questions grouped into 4 categories (i.e. general, medical, nutritional, and occupational) are obtained based on participants’ subjective reports through face-to-face interviews. The general questionnaire is subdivided to nine sections comprising demographic parameters, socioeconomic status, occupational history, fuel exposures, lifestyle, sleep and circadian rhythm, physical activity, mobile use, and pesticide exposures. The medical questionnaire consists of personal and familial medical history, medication profile, reproductive history (for women), oral hygiene, past and current substance abuse (i.e. smoking, alcohol, and drug), and questions inspecting for alopecia and gross abnormalities in limbs and torso. The information on dietary intake over a 1-year period is obtained using the Willet format of the Food Frequency Questionnaire (FFQ) [35]. The current food preparation and preservation techniques are also obtained. The occupational questionnaire consists of scheduling issues, occupational hazards, and job-related pain or disorders.

In addition, the psychological condition of participants is assessed in a self-administered manner. The dimensions of depression, anxiety, and stress are measured using the short-form version of the Depression Anxiety and Stress Scale (DASS-21) [36]. The Pittsburgh Sleep Quality Index (PSQI) is used to measure sleep quality over a 1-month time interval [37]. In addition, if the participant is in direct contact with patients, the job stress level is measured using the 35-item Hospital Stress Scale (HSS-35) [38], otherwise, the Steinmetz occupational stress questionnaire [39] is administered.

#### Satisfaction

The satisfaction checklist consists of 15 closed questions describing distinct steps of the process (i.e. advertisement, scheduling and coordination, laboratory, anthropometry, general, nutrition, medical, duration, circulation, management, physical environment, etc.). A 4-point Likert scale (weak to perfect) is used to measure subjects’ level of satisfaction. Overall satisfaction is measured by averaging the response values and results are used to send regular feedback to the PI committee, interviewers, and examiners.

### Follow-up phase

This study is aimed to obtain long-term clinical data from participants approximately 15 years after their enrollment. Phone interviews are made to collect information on changes in health status and its determinants, using the Interactive Voice Response (IVR) system in yearly intervals. If there is no answer after six calls in three different weeks, the follow-up team consisting of a nurse or physician and a sampling technician will go to the postal address of the participant and attempt to perform the interview in a face-to-face manner. Follow-up interviews consist of questionnaires to be filled out once there is an incidence of death, medical events, hospitalizations, diagnostic procedures, or therapeutic services. In addition, an outcome review team consisting of two trained internists will ultimately verify the cause of death or diagnosis of medical events. If consensus is not made, the third internist will review the documents to reach a decision. At the 5-, 10-, and 15-year time points the entire process is repeated for alive participants.

### Quality assurance

Due to the large-scale design and an extensive amount of data to be collected and processed, quality assurance (QA) is of crucial importance and depends on a variety of factors related to the study personnel and equipment. The most effective technique to achieve high-quality data is the implementation of validation rules in the data entry software (especially required check validations to spot missing points and range check validations to detect and alarm on outliers). The QA process is performed at both the local and national level. The local QA team is responsible to regularly check the age and gender distributions aiming to detect bias during the enrollment process. The prepared QA forms are also filled out regularly by the local QA team and sent to the central team. To evaluate the interviewers, voice is recorded at random, for which the interviewee is unaware, after obtaining informed consent from the interviewee. The recording can then be reviewed by the national QA team. In addition, some of the enrolled participants are invited to repeat some fixed measurements and questions, so the new and previously recorded data can be compared. It should also be noted that all devices are calibrated and controlled regularly for accurate functioning by technical agents of the companies.

## Discussion

The POCM is the first organizational cohort study aiming to determine the burden and risk factors associated with the NCDs in the northeast of Iran (with mainly Fars, Turk, and Kurd ethnicities). The POCM includes numerous variables on emerging risk factors as well as the established traditional risk factors of the NCDs. The enrollment phase with an average daily capacity of 18 participants is currently underway and will be completed in the middle of 2021.

A recent review has reported that the incidence of thyroid cancer has continuously and sharply increased worldwide [40]. In spite of considerable advances in diagnosis modalities and treatment approaches, the mortality rate due to thyroid cancer has not decreased. Environmental carcinogens accompanying the lifestyle in the industrialized countries have mainly affected the thyroid. Since the POCM is the only PERSIAN cohort center located in a metropolitan area, the PI committee decided to perform thyroid dysfunction screening as well as ultrasound examination to study the well-known or unrecognized risk factors.

Since the cardiovascular disorders are the leading cause of death in Iran, the appropriate management of classical and biological risk factors together with novel measures may represent a better approach for more accurate predictions of these disorders. Arterial stiffness is the early morphological changes of vessels leading to many cardiovascular risks. Thus, the arterial elastic properties have been introduced for risk stratification purposes in different populations. The PWV test is a non-invasive tool to evaluate the arterial system damage [41]. To the best of our knowledge, the POCM is the first study measuring the PWV indices in a large Iranian population aiming to determine regional normal ranges as well as its prognostic significance for cardiovascular mortality.

The most notable statistic in baseline assessment is the high prevalence (64.9%) of overweight defined as body mass index of greater than 25 kg/m^2^. Rapid urbanization combined with changes in dietary intake and physical activities have led to an increase in overweight and obesity in developing countries [42]. This is important information to direct services, training, and programs when deciding on how to manage limited resources for obesity prevention and treatment among personnel who are responsible for patient care.

In comparison with other PERSIAN cohort centers [43–45], the POCM seems to be the most comprehensive one performing 16 examinations and tests aiming to detect a large number of NCD events (especially cardiovascular disorders). Although the average time of the enrollment process is rather long (3 h and 45 min), the increasing number of volunteers shows an acceptable level of satisfaction with respect to a wide range of examinations.

The following actions are taken into account to improve the quality of data: 1) Trained personnel were responsible for gathering and entering data according to the study protocol aiming to minimize inter-observer variability, 2) The data entry software included range check validations to avoid outliers as far as possible, 3) The field supervisor is responsible for regular data checks for missing data, and 4) The local and central quality assurance teams perform regular quality control checks.

We note the following limitations: First, inclusion of the MUMS personnel affects the generalizability of the results to the general population concerning the education level, health literacy, etc. However, the PI committee has started negotiations with other organizations in Mashhad (e.g. Mashhad municipality, Ferdowsi University of Mashhad, etc.) to provide a more representative sample of the northeastern population of Iran. Moreover, retired employees are also invited to include elderly individuals who have worked at least 25 years in the organizations. Second, since the participants have employment relationship with the MUMS, and substance abuse (especially alcohol) is not acceptable according to religious and cultural backgrounds, the underestimation of such statistics is very likely. However, the participants are ensured about the confidentiality/anonymity of their data at the beginning of the process. Third, the lack of fully standardized risk factor measurement guideline in some examinations (e.g. imaging studies) may affect the results.

In conclusion, the POCM is the first organizational cohort study in a metropolitan city of Iran aiming to provide a large data repository on the prevalence and risk factors of the NCDs in a developing country to pave the way for future national and international research cooperation. Researchers interested in a collaborative study are invited to contact the POCM principal investigator, Saeid Eslami, at S.eslami.h@gmail.com.

## Data Availability

The datasets used and/or analyzed during the current study are available from the corresponding author on reasonable request.

## Abbreviations

ATS/ERS: American Thoracic Society/European Respiratory Society
BP: Blood Pressure
CBP: Central Blood Pressure
CC: Cranial-Caudal
DASS: Depression Anxiety and Stress Scale
EPIC: European Prospective Investigation into Cancer and Nutrition
FFQ: Food Frequency Questionnaire
FOB: Fecal Occult Blood
FVC: Forced Vital Capacity
HSS: Hospital Stress Scale
INCDC: Iran established the Iranian NCD committee
IOP: Intra Ocular Pressure
IVR: Interactive Voice Response
MLO: Mediolateral-Oblique
MOHME: Ministry of Health and Medical Education
MUMS: Mashhad University of Medical Sciences
NAFLD: Non-Alcoholic Fatty Liver Disease
NCD: Non-Communicable Disease
OCTA: Optical Coherence Tomography Angiography
PCID: PERSIAN Code Identifier
PERSIAN: Prospective Epidemiological Research Studies in IrAN
PI: Principal Investigator
POCM: PERSIAN Organizational Cohort study in MUMS
PSQI: Pittsburgh Sleep Quality Index
PWV: Pulse Wave Velocity
QA: Quality Assurance
TPSA: Total Prostate-Specific Antigen
TSH: Thyroid Stimulating Hormone
YLD: Years Lived with Disability
